# Management of antibacterial therapy of infectious and inflammatory diseases of the urinary tract in children and regional peculiarities during the COVID-19 pandemic

**DOI:** 10.25122/jml-2021-0293

**Published:** 2022-05

**Authors:** Volodymyr Volodymyrovych Bezruk, Igor Dmytrovych Shkrobanets, Oleksii Serhiiovych Godovanets, Oleksandr Hryhorovych Buriak, Olga Ivanivna Pervozvanska, Ludmila Mykhailivna Honcharuk, Nina Ivanivna Voytkevich, Olena Victorivna Makarova, Oksana Ivanivna Yurkiv, Michael Ivanovych Sheremet, Mykhailo Mykhailovich Hresko, Mariya Ivanivna Velia, Svyatoslava Vasylivna Yurniuk, Maryna Dmytrivna Hresko, Tetiana Sergiivna Bulyk, Larysa Vasylyvna Rynzhuk

**Affiliations:** 1. Department of Pediatrics, Neonatology and Perinatology Medicine, Bukovinian State Medical University, Chernivtsi, Ukraine; 2. Department of Medical and Organizational Management, National Academy of Medical Sciences of Ukraine, Kiev, Ukraine; 3. Department of Nephrology, Pediatric Clinical Hospital, Chernivtsi, Ukraine; 4. Department of Internal Medicine, Bukovinian State Medical University, Chernivtsi, Ukraine; 5. Department of Foreign Languages, Bukovinian State Medical University, Chernivtsi, Ukraine; 6. Department of Care for Patients and Higher Nursing Education, Bukovinian State Medical University, Chernivtsi, Ukraine; 7. Surgery Department No.1, Bukovinian State Medical University, Chernivtsi, Ukraine; 8. Department of Pharmacy, Bukovinian State Medical University, Chernivtsi, Ukraine; 9. Department of Obstetrics and Gynecology, Bukovinian State Medical University, Chernivtsi, Ukraine

**Keywords:** urinary tract infections, COVID-19, antibiotic resistance, children

## Abstract

Urinary tract infections (UTIs) remain an urgent issue in clinical pediatrics. Empirical selection of antibacterial therapy becomes more complicated, and antibacterial drug indication is not always clinically substantiated. This study aimed to compare the antibacterial susceptibility pattern of the main group of urinary tract infectious agents from 2009–2016 with intermediate results from 2020–2021, during the COVID-19 pandemic, among children in the Chernivtsi region. Urine samples were collected from 3089 children (0–17 years old) treated at the health care institutions in the Chernivtsi region (2009–2016). The clinical-laboratory examination of 177 children (0–17 years old) was carried out from 2020 to 2021. The children received specialized medical care at the Department of Nephrology. Preliminary data of regional monitoring (2020–2021) are not considerably different from the previous regional susceptibility of antibiotics: to penicillin (p<0.01), ІІ-ІІІ generation cephalosporin (p<0.01); an increased resistance to levofloxacin (χ^2^=4,338; p<0.01), tetracycline – χ^2^=7,277; p<0.01; doxycycline – χ^2^=5,309; p<0.01) and imipenem – χ^2^=5,594; p<0.01). The data obtained did not explain an increased resistance to fluoroquinolones completely (ofloxacin, pefloxacin, ciprofloxacin), except for levofloxacin (χ^2^=4,338; p<0.01). A reliable difference of susceptibility of tetracycline group was registered (tetracycline – χ^2^=7,277; p<0.01; doxycycline – χ^2^=5,309; p<0.01). Furthermore, there was a regional increase in some UTI-pathogen strains resistant to carbapenems (imipenem – χ^2^=5,594; p<0.01). The use of antibiotics from the group of penicillins and II-III generation cephalosporins as the starting antibacterial therapy for STIs during the COVID-19 pandemic should be justified. A regional increase (2020–2021) of some uropathogenic strains resistant to carbapenems administered to treat severe bacterial infections requires their exclusively designated purpose in everyday pediatric practical work.

## INTRODUCTION

Antibiotic resistance to the main agents of infectious diseases is one of the greatest problems in modern medicine [1–3]. Antibiotics are widely used in modern medicine since these increase life expectancy, especially in a significantly vulnerable group of older people who have been disproportionately affected by the current COVID-19 pandemic. Antibiotic resistance both during and after these events is well recognized [4–6].

The use of antibacterial therapy is not always clinically justified according to antibacterial sensitivity. Urinary tract infections (UTIs) remain an urgent issue in clinical pediatric sections. Empirical selection of antibacterial therapy becomes more complicated, and administration of an antibacterial drug is not always clinically substantiated [7–16].

## MATERIAL AND METHODS

This study aimed to compare the susceptibility of antibacterial drugs to the main groups of urinary tract infectious agents from 2009–2016 with intermediate results from 2020–2021 among children in the Chernivtsi region during the COVID-19 pandemic. We studied the etiological structure of uropathogens – UTI pathogens among children in the Chernivtsi region from 2009–2016 and 2020–2021. Urine samples were collected from 3089 children (0–17 years old) in the Chernivtsi region (2009–2016). Clinical-laboratory examination of 137 children (0–17 years old) who received specialized medical care at the Department of Nephrology during 2020–2021 was performed. There were 102 (74.45%) children with infectious-inflammatory urinary tract diseases (the diagnosis was made according to ICD-10: No.10-11.1) as follows: kidney infection including No.10 acute tubulointerstitial nephritis – 52 patients; No.11 chronic tubulointerstitial nephritis – 21 patients; No.11.1 chronic obstructive pyelonephritis – 8 patients; No.30.0 acute cystitis – 10 patients; No.30.1 chronic cystitis – 11 patients) and 35 (25.55%) children with non-infectious diseases of the urinary tract (according to ICD-10: N00 acute nephrotic syndrome – 6 patients; No.03 chronic nephrotic syndrome – 5 patients; No.04 nephrotic syndrome – 10 patients; No.15; other renal tubulointerstitial diseases – 3 patients; No.18 chronic renal failure – 6 patients; No.39.2 orthostatic proteinuria, not specified – 2 patients; R30 pain associated with urination – 1 patient; R30.1 tenesmus of the urinary bladder – 1 patient; R32 enuresis, not specified – 1 patient).

## RESULTS

Preliminary intermediate data of the regional monitoring (2020–2021) of antibiotic susceptibility to UTI-pathogens and *Enterobacterales* family in particular as leading etiological agents among children in the Chernivtsi region during the COVID-19 pandemic did not considerably differ from previous screening (2009–2016), namely to penicillin (p<0.01), and ІІ-ІІІ generation cephalosporin (p<0.01) [17, 18].

The data obtained from 2020 to 2021 did not reveal increased resistance to fluoroquinolones completely (ofloxacin, pefloxacin, ciprofloxacin), except levofloxacin (χ^2^=4,537; p<0.01). A reliable difference of sensitivity to the tetracycline group was registered (tetracycline – χ^2^=7,307; p<0.01; doxycycline – χ^2^=5,369; p<0.01). A regional increase of some UTI-pathogen strains resistant to carbapenems (imipenem – χ^2^=5,613; p<0.01) was also registered.

Preliminary intermediate results from the clinical-laboratory examination of 137 children (0–17 years) in 2020–2021, who received specialized medical care at the Department of Nephrology, found a reliable difference in the etiological structure of the microbiota in the urine of children with infectious-inflammatory diseases of the urinary tract: *gram-positive cocci* – n=21 (21.0%), p=0.042; *enterobacteria* – n=26 (25.0%), p=0.012; *resident microbiota* – n=17 (17.0%), p=0.000 compared with isolation of UTI-pathogens in case of non-infectious disease of the urinary tract in children: *gram-positive cocci* – n=5 (14%); *enterobacteria* – n=2 (6.0%); *resident microbiota* – n=26 (74.0%) (Table 1).

**Table 1:** The etiological spectrum of UTI-pathogens in children with diseases of the urinary tract examined during 2020–2021.

Structure of the etiological spectrum of UTI-pathogens isolated	Infectious-inflammatory diseases of the urinary tract (n=102)		Non-infectious-inflammatory diseases of the urinary tract (n=35)		p
	abs., n	%	abs., n	%	
**Lack of growth**	38	37%	5	14%	0.012
***Gram-positive cocci***	21	21%	2	6%	0.042
***Enterobacteria***	26	25%	2	6%	0.012
***Resident microbiota***	17	17%	26	74%	0.000

## DISCUSSION

Antibiotic resistance is an urgent problem, and it has become relevant during the coronavirus infection pandemic. A rational approach to the choice of antibacterial therapy during the COVID-19 pandemic improves the clinical picture, reduces the cost of treatment for each patient, and helps maintain the sensitivity of pathogens in the long term [19].

The intermediate results of regional monitoring (2020–2021) of antibiotic resistance among children in the Chernivtsi region raise certain concerns about the increase of some uropathogenic strains resistant to carbapenems (imipenem – χ^2^=5,613; p<0.01). This is relevant given the growing resistance to carbapenems during the COVID-19 pandemic [20, 21].

Carbapenems are used to treat severe bacterial infections caused by gram-negative causative agents resistant to antibacterial therapy. Nowadays, the treatment of gram-negative infections, including those with multiple pharmacological resistance, should be based on information about regional antibiotic susceptibility and the local epidemiological picture. The presence of carbapenems in the doctor's arsenal is an important support in the context of pandemic multidrug resistance as it requires investigation of specific algorithms and improvement tactics to administer antibacterial drugs. Therefore, major factors to consider in administering carbapenems are cooperation between doctors and microbiologists, finding the focus of infection before empirical therapy indication, and considering the synergic effects of antibiotics for a combined therapy [22, 23].

## CONCLUSIONS

Administration of potentially pathogenic therapy (*e.g*., antibiotics from the group of penicillins and II-III generation cephalosporins as starting antibacterial therapy in children) should be carefully managed due to recent increases in regional antibiotic resistance of uropathogens. Bacterial strains resistant to carbapenems used to treat severe infections during the COVID-19 pandemic also require strict risk-benefit consideration in everyday pediatric clinical practice. Dynamic monitoring and surveillance of regional antimicrobial resistance should be enforced and used to inform clinical practice and contain the phenomenon.

## References

[1] Pedroso MM, Selleck C, Enculescu C, Harmer JR (2017). Characterization of a highly efficient antibiotic-degrading metallo-β-lactamase obtained from an uncultured member of a permafrost community. Metallomics.

[2] Vivas R, Barbosa AAT, Dolabela SS, Jain S (2019). Multidrug-Resistant Bacteria and Alternative Methods to Control Them: An Overview. Microb Drug Resist.

[3] Quillaguamán J, Guzmán D, Campero M, Hoepfner C (2021). The microbiome of a polluted urban lake harbors pathogens with diverse antimicrobial resistance and virulence genes. Environ Pollut.

[4] Mahmoudi H (2020). Bacterial co-infections and antibiotic resistance in patients with COVID-19. GMS Hyg Infect Control.

[5] Langford BJ, So M, Raybardhan S, Leung V (2021). Antibiotic prescribing in patients with COVID-19: rapid review and meta-analysis. Clin Microbiol Infect.

[6] Livermore DM (2021). Antibiotic resistance during and beyond COVID-19. JAC Antimicrob Resist.

[7] Karmazyn BK, Alazraki AL, Anupindi SA, Dempsey ME (2017). Expert panel on pediatric imaging: ACR appropriateness criteria, urinary tract infection-child. J. Am. Coll. Radiol.

[8] Korbel L, Howell M, Spencer JD (2017). The clinical diagnosis and management of urinary tract infections in children and adolescents. Paediatr. Int. Child Health.

[9] Balighian E, Burke M (2018). Urinary tract infections in children. Pediatr. Rev.

[10] Visuri S, Jahnukainen T, Taskinen S (2018). Prenatal complicated duplex collecting system and ureterocele-Important risk factors for urinary tract infection. J Pediatr Surg.

[11] Öztürk R, Murt A (2020). Epidemiology of urological infections: A global burden. World J. Urol.

[12] Kaur R, Kaur R (2021). Symptoms, risk factors, diagnosis and treatment of urinary tract infections. Postgrad Med J.

[13] Meštrović T, Matijašić M, Perić M, Čipčić Paljetak H (2020). The Role of Gut, Vaginal, and Urinary Microbiome in Urinary Tract Infections: From Bench to Bedside. Diagnostics.

[14] Bonkat G, Pickard R, Bartoletti R (2018). Urological Infections. EAU Guidelines.

[15] Shaikh N, Shope MF, Kurs-Lasky M (2019). Urine specific gravity and the accuracy of urinalysis. Pediatrics.

[16] Boon HA, Van den Bruel A, Struyf T, Gillemot A (2021). Clinical Features for the Diagnosis of Pediatric Urinary Tract Infections: Systematic Review and Meta-Analysis. Ann Fam Med.

[17] Bezruk VV, Bezruk TO, Babiy OR, Sokolnyk SO (2017). Regional monitoring of the urinary tract infections etiological spectrum pathogens in the child population in Chernivtsi region: dynamic changes, age, gender, administrative and territorial characteristics. Zaporozhye Medical Journal.

[18] Bezruk VV, Bezruk TO, Stegnitska LV, Sokolnyk SO (2017). Regional monitoring of the urinary tract infections causative agents antibiotic resistance in the child population of the Chernivtsi region. Zaporozhye Medical Journal.

[19] López-Jácome LE, Fernández-Rodríguez D, Franco-Cendejas R, Camacho-Ortiz A (2022). Increment Antimicrobial Resistance During the COVID-19 Pandemic: Results from the Invifar Network. Microb Drug Resist.

[20] Mamishi S, Mahmoudi S, Naserzadeh N, Hosseinpour Sadeghi R (2019). Antibiotic resistance and genotyping of gram-negative bacteria causing hospital-acquired infection in patients referred to Children's Medical Center. Infect Drug Resist.

[21] Despotovic A, Milosevic B, Cirkovic A, Vujovic A (2021). The Impact of COVID-19 on the Profile of Hospital-Acquired Infections in Adult Intensive Care Units. Antibiotics (Basel).

[22] Dlewati MM, Aung PP, Park K, Rodriguez JA, Poon KK (2021). Meropenem-Resistant Pandoraea Pneumonia in a Critically Ill Patient With COVID-19. Cureus.

[23] Baba H, Kanamori H, Seike I, Niitsuma-Sugaya I (2021). Multiple Secondary Healthcare-Associated Infections Due to Carbapenem-Resistant Organisms in a Critically Ill COVID-19 Patient on Extensively Prolonged Venovenous Extracorporeal Membrane Oxygenation Support-A Case Report. Microorganisms.

